# Use of ultrasound guided single shot costotransverse block (intertransverse process) in breast cancer surgery: a prospective, randomized, assessor blinded, controlled clinical trial

**DOI:** 10.1186/s12871-022-01651-3

**Published:** 2022-04-18

**Authors:** Hakan Aygun, Ilker Kiziloglu, Nilgun Kavrut Ozturk, Haydar Ocal, Abdullah Inal, Leyla Kutlucan, Edip Gonullu, Serkan Tulgar

**Affiliations:** 1 Department of Anesthesiology, Bakircay UniversityFaculty of Medicine Cigli Training and Research Hospital, Izmir, Turkey; 2 Department of General Surgery, Bakircay UniversityFaculty of Medicine Cigli Training and Research Hospital, Izmir, Turkey; 3 Department of Anesthesiology, University of Health Science Faculty of MedicineAntalya Training and Research Hospital, Antalya, Turkey; 4 Department of General/Oncological Surgery, Bakircay University Faculty of MedicineCigli Training and Research Hospital, Izmir, Turkey; 5 Department of Anesthesiology/Algology, Bakircay UniversityFaculty of MedicineCigli Training and Research Hospital, Izmir, Turkey; 6 Department of Anesthesiology, Samsun University Faculty of Medicine, Samsun Training and Research Hospital, Samsun, Turkey

**Keywords:** Breast cancer, Postoperative Pain, Nerve Block

## Abstract

**Background:**

Ultrasound guided costotransverse block (CTB) is a relatively new “peri-paravertebral” block that has been described recently. It has been previously reported that CTB, administered with a single high-volume injection, provides effective analgesia in breast conserving surgery. In this study we evaluated the effect of CTB when used in breast cancer surgery.

**Methods:**

Seventy patients due to undergo breast cancer surgery were included in this blinded, prospective, randomized, efficiency study. Patients were randomized into two equal groups (CTB group and control group) using the closed envelope technique. All patients underwent general anesthesia. In addition to standard analgesia methods, patients in group CTB also received CTB block while the remaining (control group) did not. Numeric rating (pain) scores and opioid consumption was compared between the two groups.

**Results:**

Opioid consumption in all time frames and pain scores at 1st and 3rd hours only were found to be significantly lower in Group CTB when compared to the control group.

**Conclusions:**

Ultrasound guided CTB improves analgesia quality in breast cancer surgery.

**Trial registration:**

Clinicaltrials Registration ID: NCT04197206, Registration Date: 13/12/2019.

**Supplementary Information:**

The online version contains supplementary material available at 10.1186/s12871-022-01651-3.

## Background

Breast surgeries are common as breast cancer is a very common oncological disease [1]. Although increased use of more non-invasive breast conserving surgical procedures leading to a better recovery period when compared to more conventional techniques, the management of postoperative pain is still an important part of an anesthesiologists practice [1, 2]. With the increased use of ultrasonography (USG), interfascial plane blocks have become an important part of multimodal analgesia for the management of both acute and, where necessary, chronic pain following breast surgery [3]. Randomized clinical studies have shown that pectoral nerve blocks (PECs I-II), parasternal blocks, erector spinae plane blocks, superficial and deep serratus blocks and rhomboid intercostal blocks are effective for postoperative analgesia following breast surgery [3–5].

USG guided costotransverse block (CTB), recently described by Nielsen et al. [6], is a relatively new “peri-paravertebral” block. At this point the area of local anesthesia administration is more superficial to a thoracic paravertebral block (TPV). The injection point lies in the thoracic intertransverse tissue complex. Multiple injections are utilized with the needle placed at the costotransverse junction just before it reaches the cranial portion of the neck of the underlying rib [6]. We have previously reported effective analgesia in breast conserving surgery with CTB applied from the level of the 4th rib using a high volume single injection [7].

This study aimed to determine the effect of single injection CTB on analgesic requirement and pain scores when used as a part of a multimodal analgesia plan in patients having breast cancer surgery.

## Methods

### Study design

This randomized, controlled, double blinded study was conducted between January 2020 and May 2020 following local ethical committee approval (Approval No: 20/11/2019-279) and clinicaltrials.gov registration (Registration No: NCT04197206 Registration Date: 13/12/2019) and in accordance with Consolidating Standards of Reporting Trials (CONSORT 2010) guidelines.

American Society of Anesthesiologists (ASA) class I-II female patients (18-80 years) were included in the study. Those scheduled to undergo TPV block or other regional anesthesia techniques were excluded from the study. In addition, the following exclusion criteria were used: morbid obesity (> 100 kg, BMI > 35 kg / m2), ASA III- IV, skin infection at puncture site, known study drug allergies, coagulation disorders (abnormal INR, thrombocytopenia etc.) and recent use of analgesic/steroid drugs.

### Randomisation and groups

**Fig. 1:**
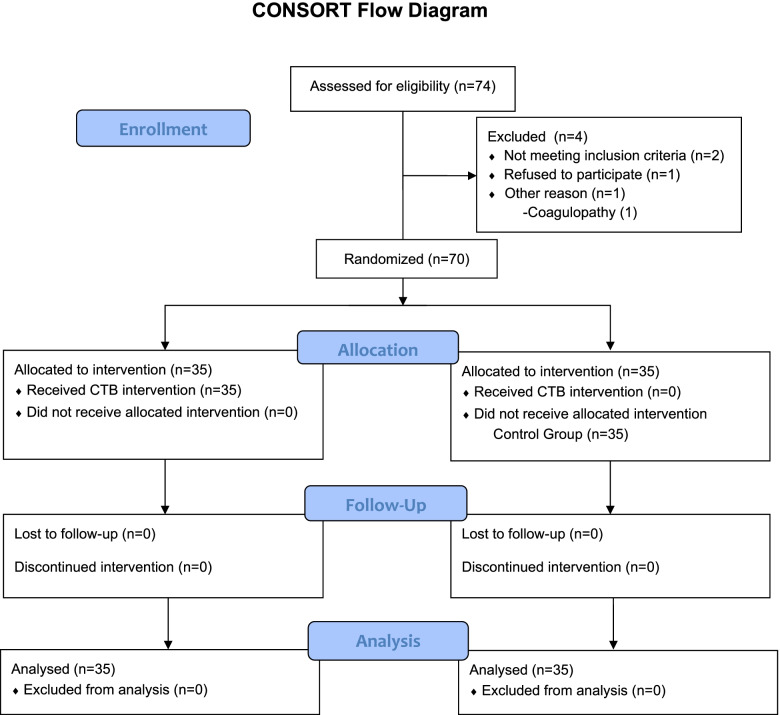
CONSORT flow diagram of study

Patients were randomized, using the closed envelope technique, into the CTB or control group in the preoperative waiting area (HO). There were an equal number of envelopes containing CTB and control group assignments. Each patient received a random ID number that was used at all phases of the study. Patients in the control group underwent a standard analgesia plan. Additionally, patients in the CTB group also received a single sided CTB block following anesthesia induction. All blocks were performed under ultrasound guidance by an anesthesiologist experienced in regional anesthesia applications (HA). Postoperative followup was performed by two authors that were blinded to the randomization and block application processes (LK, AI). These assessors of postoperative pain and analgesia use were not aware of which group the patients were assigned to, and used the randomization ID for all data collection. The CONSORT chart of this assessor blinded study is shown in Fig. 1.

### USG guided CTB block

**Fig. 2:**
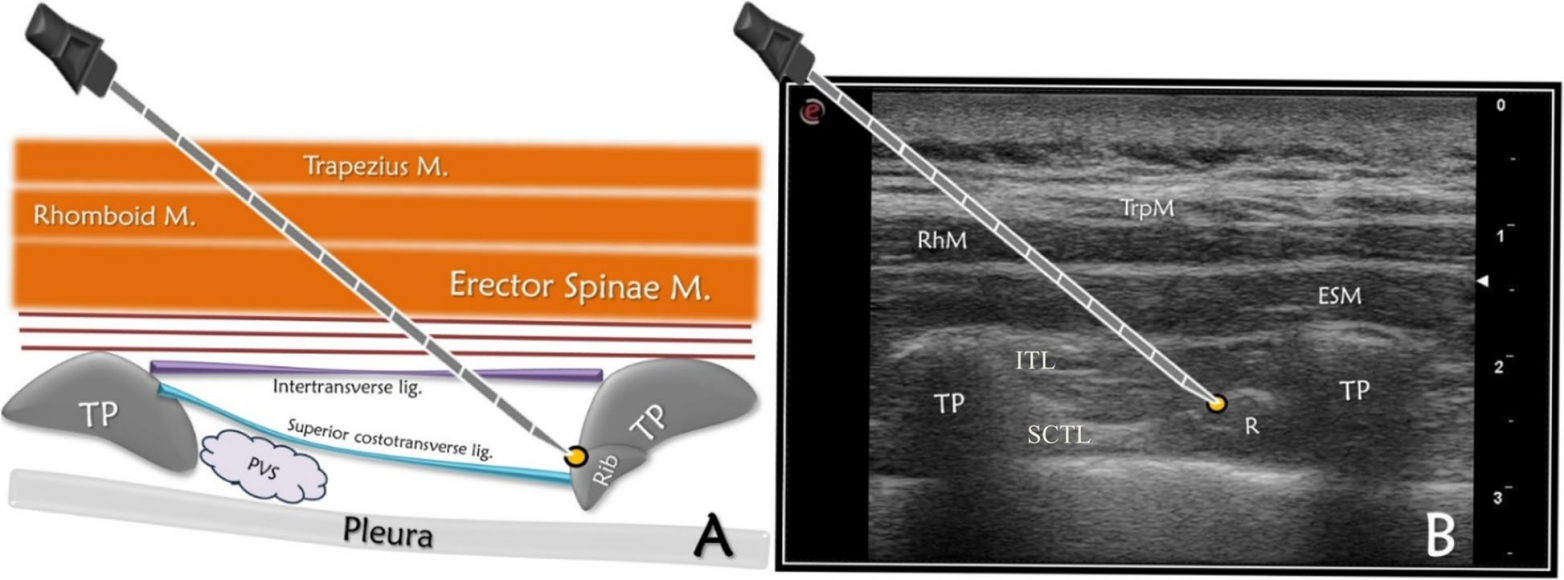
Ultrasound anatomy of costotransverse block

Patients in both groups received premedication of 0.03 mg/kg of IV midazolam in the preoperative waiting room. For patients in the CTB group: after anesthesia and following skin prep, a high frequency linear USG transducer was placed 2-2.5 cm lateral to the spinous process of the 4th thoracic vertebra. CTB block was performed unilaterally on the ipsilateral side of surgery. The skin and subcutaneous layers, trapezius and erector spinae muscles, third and fourth transverse processes, intertransverse and superior costotransverse ligaments (SCTL) as well as the pleura were placed in the field of view. A needle was advanced parallel to the SCTL, stopping immediately before reaching the cranial part of the fourth rib. Here, a mixture of 0.5% bupivacaine (15 mL) and 2% lidocaine (10 mL) was administered (Fig. 2). We used the in-plane technique and the needle advanced from cephalad caudally.

### General anesthesia and perioperative analgesia management

The same general anesthesia plan was applied in both groups. Propofol (2-3 mg/kg), fentanyl (1.5 μg/kg) and rocuronium bromide (0.6 mg/kg) were used for induction and following endotracheal intubation anesthesia maintained by utilizing sevoflurane (0.6-1 MAC) and remifentanil infusion (0.08-0.2 mcg/kg/min). Paracetamol 1 g IV infusion and dexketoprofen trometamol 50 mg i.v. was administered at least 30 min before the end of surgery. After extubation patients were moved to the recovery room where patients were started on PCA. The PCA consisted of 0.5 mg/mL of morphine administered with no basal infusion followed by boluses of 1 mg each and lockout of 20 min after each application. Paracetamol was applied every 8 h for the first 24 h. Postoperative pain was measured at different time points (at hours 1, 6, 12 and 24 postoperatively) using the numeric rating scale (NRS).

### Outcome measures

Primary and secondary outcomes of this study were opioid requirement (first postoperative 24 h) and NRS scores at rest measured at the aforementioned five different time frames. The time for first requirement of analgesic and presence of nausea and/or vomiting was also recorded.

### Statistical analyses

A pilot study of ten patients using the same study design was conducted before this study. The pilot study revealed 24 h morphine use to be 4 ± 2.30 mg vs 7.2 ± 1.31 mg in the CTB and control groups, respectively. Using this data, the minimum required sample size was calculated to be 13 participants for each group (alpha 0.05, beta 0.10 and power of 0.95). However, when considering the sample sizes of first reports of newly defined fascial plane blocks in literature [2, 4, 5] and also considering the analysis of secondary outcomes, a decision was made to increase the number of participants in each group to 35, also taking into consideration any possible loss to followup.

SPSS for Windows, Version 16.0. (SPSS Inc., Chicago, USA) was used for statistical analysis. The Kolmogorov-Smirnov test was used to test for normality of data. Continuous variables were expressed as mean ± standard deviation, and median (25th–75th percentiles). Independent t-test was utilized for univariate analysis of continuous variables of equal variance. Non-normally distributed data was evaluated using Mann Whitney U test. Ratios were compared with Chi-square test. Where categorical variables were concerned the Fisher’s exact test was utilized. Kaplan-Meier analysis and Wilcoxon test was used when comparing time to first analgesia. Statistical significance was accepted as *p* < 0.01 for NRS scores (after Bonferroni correction) and *p* < 0.05 for all other comparisons.

## Results

Seventy patients from 74 recruits were included in the study. The CONSORT diagram is shown in Fig. 1. Average age, weight, height, body mass index (BMI), surgical times, surgery types and block performance time were similar between groups and are shown in Table 1.

**Table 1:** Patient demographics, surgical times/types and statistical evaluation

	CTB Group (n:35)	Control Group (n:35)	***P***
**Age (years)**	47.74 ± 13.36	54.23 ± 9.74	0.023
**Weight (kg)**	70 ± 10.05	72.83 ± 11.73	0.282
**Height (cm)**	162 ± 6.35	164 ± 5.42	0.161
**Body Mass Index**	26.83 ± 4.17	27.17 ± 4.49	0.743
**Surgical Time (min)**	93.26 ± 37.87	93.37 ± 32.78	0.989
**Surgery Type 1 vs 2**^a^	15/20	17/18	0.631
**Block time (m)**	7.83 ± 1.32	N/A	N/A

^a^Surgery Type: 1-modified radical mastectomy, 2-simple mastectomy + sentinel lymph node biopsy (SLNB) + (axillary curettage if required). Surgery types are expressed as number of patients whereas all other data is expressed as mean ± standard deviation

**Fig. 3:**
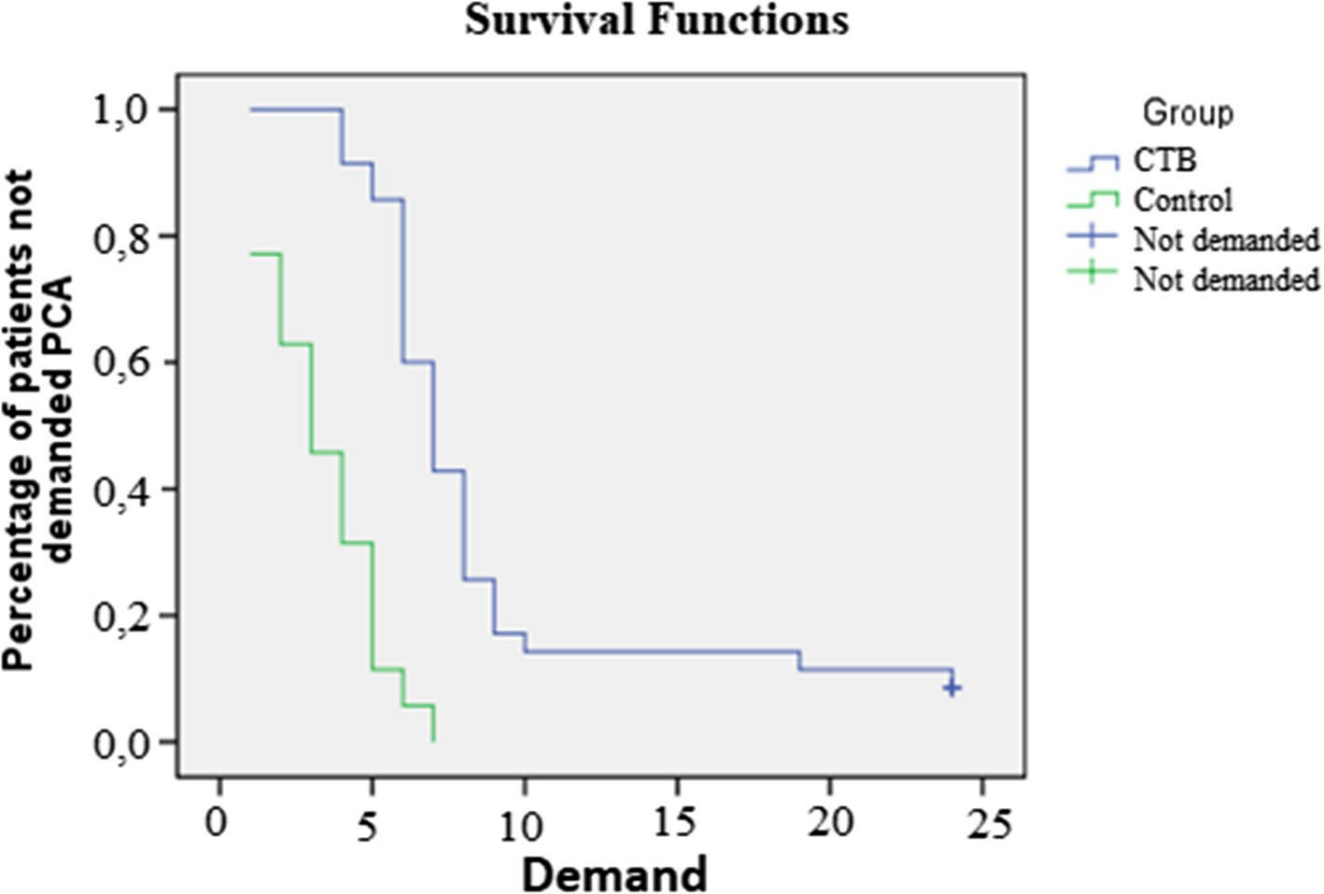
Kaplan-Meier analysis of the first opioid requirement of patients

Morphine consumption and NRS scores at different time frames as well as time to first PCA demand is shown in Table 2. There was extremely statistically significantly less morphine consumption in the CTB group in all time frames except for the first hour No patients required opioid during the postoperative first hour. While the cumulative 24 h morphine consumption was 7.51 ± 2.11 mg in the control group it was 4.26 ± 2.39 mg in the CTB group. First analgesia requirement was at an average of 6.34 ± 3.41 h in the CTB group compared to 3.34 ± 1.85 h in the control group (*p* < 0.001). The first opioid requirements of the patients are presented in Fig. 3. NRS scores were statistically significantly lower in the CTB group when compared to the control group at the 1st and 3rd hours and similar at other time frames.

**Table 2:** Average NRS scores and opioid consumption during the first 24 h, and time to the first PCA demand

	CTB (n:35)	Control (n:35)	***P***
**NRS**			
1st Hour	2 (1-2.5)	3 (3-4)	***<0.001***
3th Hour	3 (2-3)	4 (3-5)	***<0.001***
6th Hour	3 (2-3)	4 (3-4)	*0.046*
12th Hour	3 (2-3)	3 (3-3)	*0.328*
24th Hour	2 (0-2)	2 (0-2)	*0.898*
**Cumulative Morphine (mg)**			
1st Hour	0 (0-0)	0 (0-1.5)	Null
3th Hour	0 (0-0)	2 (0-3)	***<0.001***
6th Hour	1 (0-2)	4 (2-5)	***<0.001***
12th Hour	4 (2-5)	6 (5.5-8)	***<0.001***
24th Hour	4 (2-6)	7 (6-9)	***<0.001***
**Time to First PCA demand (h)**			
	6 (5.5-8)	3 (2-5)	***<0.001***

Data are expressed as median (percentiles 25–75). *p* values were italicized and *p* values that are written in bold represent statistical significance

## Discussion

Our study demonstrated that in those undergoing surgery for breast cancer, single level USG guided unilateral CTB significantly decreases 34 h opioid consumption and pushes back the time for first requirement of analgesia. NRS scores were also relatively lower in the first postoperative hours for the CTB group. As far as we are aware; this is the first study to investigate the efficacy of CTB on the quality of postoperative analgesia, and our findings support those published in our earlier case report [7].

For many years, PVB has been utilized for postoperative analgesia in breast surgery and despite some debate is accepted as the gold standard [8]. However, its high complication rate, the possibility puncture of the pleura and nerve/vascular structures in the paravertebral area leading to sympathetic blockage and the frequent requirement for multiple injections has meant that PVB has not gained popularity amongst clinicians for use as an analesia technique after breast surgery [9, 10]. The search for safer blocks continues and pectoral nerve blocks (PECs I-II), serratus plane blocks, erector spinae plane blocks (ESPB) and rhomboid intercostal nerve blocks are some facial plane blocks that have been shown through clinical trials to be effective in for postoperative analgesia following breast cancer surgery [2, 5, 11].

In ESPB, injection of LA occurs between the erector spinae muscle and the transverse process in the interfascial plane. It is thought that the effect mechanism of ESPB in breast surgery is spread of the LA anteriorly [5]. Although studies have reported that ESPB is almost as effective as PVB for analgesia in this patient group [12], the statistically significant effect of ESPB on reducing postoperative opioid requirement has been deemed as “clinically non-important” by authors of a meta analysis [13]. Studies demonstrating the opposite have also been reported [14]. Also, limited sensorial blockage without spread to the paravertebral space in ESPB and cadaveric and radiological studies reporting differing LA spread have led to different modifications and some practitioners have turned to previously defined peri-paravertebral blocks [6, 15–17].

In CTB, the aim of LA is the thoracic intertransverse tissue complex. Multiple injections are utilized with the needle placed at the costotransverse junction just before it reaches the cranial part of the neck of the underlying rib. The intertransverse tissue complex has been the area of consideration for many blocks but their nomenclature has caused confusion in academic studies. The recently published ASRA-ESRA Delphi Consensus Study stated that there was weak or no consensus for the midpoint midpoint transverse process to pleura (MTP), subtransverse process intraligamentary plane (STIL), costotransverse foramen plane (CTF), and the multiple injection costotransverse (MIC) blocks [18]. The study further stated that all blocks were similar in lieu of the anatomic location of injection sites and therefore “intertransverse process (ITP) block” should be considered as new nomenclature. In accordance with this study, we found it appropriate to use the “intertransverse process block” name, although we did use the name “CTB “frequently in our ethical board application and ClinicalTrial registration. To our knowledge this is the first study to use this new nomenclature.

In CTB, the application point is deeper than ESPB yet more superficial compared to PVB. Therefore CTB may be safer than PVB and have a higher success rate when compared to ESPB. However it should be noted that the innervation of the breast is highly complex [19] with the pectoral nerves also playing an important role, meaning the thoracic intercostal nerve block alone may be inadequate or ineffective.

We did not observe any complication related to CTB in our study. However, many complications including pneumothorax have been reported secondary to ESPB [15, 20], a block performed at a similar anatomic location. In regional anesthesia applications, the practitioner’s experience is frequently a predictor of and preventative factor for development of complications. CTB is more difficult to perform and deeper when compared to ESPB, yet easier and more superficial to PVB. The primary priority should be the patient’s safety, and the practitioner should choose a technique based on his own experience. Studies examining the learning curves in interfascial plane blocks and peri-paravertebral blocks applied in breast cancer surgeries are also required.

In the study by Nielsen et al. in which CTB was first defined, the authors reported results of multiple injections performed at different levels [6]. Thereafter, we reported that high volume LA performed from a single level (T4) lead to effective analgesia in breast cancer surgery. Furthermore, 15 mL of methylene blue administered at T4 level in a cadaveric study demonstrated spread of methylene blue to both the ventral rami and relatively lower spread to the dorsal rami in CTB, reported as costotransverse foramen block [21]. In line with current literature and our clinical experience, CTB was performed as a single-level high volume administration in our study. It may be useful to conduct comparative studies in terms of different volumes, levels and techniques.

Our study has several limitations. Although we performed sensorial analysis in our previous study, no routine sensorial analysis or block mapping was performed in our current study. Also, a study methodology that utilized a sham group in place of a control group would have meant our study was also double blinded. Another limitation is that pain density was evaluated using NRS only evaluated at rest. Pain levels at arm abduction could have revealed additional important findings. We found that NRS scores were relatively higher in the control group, and this is not a desirable situation in a regional anesthesia study. However, no patient experienced unbearable pain in our study. Although we could have designed our study to compare CTB with another regional analgesia technşqye, our aim was to primarily and firstly report its potential analgesic effectiveness when combined with multimodal analgesia techniques.

In this study we have demonstrated that CTB can be used effectively in those undergoing breast cancer surgery. However, CTB requires comparison with PVB, ESPB and other interfascial blocks. Also, the ideal LA volume, volume-dermatome association and LA pharmacodynamics still require determination or definition for CTB with comparison of the same parameters in PVB and other fascial blocks. Although the distribution of types of surgery was similar in this study, a controlled study evaluating more homogeneous groups would have more generalizable results.

## Conclusion

USG guided single shot CTB (intertransverse process block) improved the quality of analgesia and statistically significantly decreased opioid requirement in patients undergoing breast cancer surgery when compared to a control group. Studies evaluating different volumes and levels of injection in similar and different surgical procedures and their comparison with other block types are required.

## Supplementary Information

**Additional file 1.**

## Data Availability

The datasets used and analysed during the current study are available from the corresponding author on reasonable request.

## Abbreviations

CTB: Costotransverse block
ASA: American Society of Anesthesiologists
NRS: Numeric Rating Scores
PCA: Patient Controlled Analgesia
PECs: Pectoral nerve blocks
USG: Ultrasonography
TPV: Thoracic paravertebral block
CONSORT: Consolidating standards of reporting trials
SCTL: Superior costotransverse ligament
LA: Local anesthetic
MAC: Minimum alveolar concentration.
BMI: Body mass index
ESPB: Erector spinae plane block
MTP: Midpoint transverse process to pleura
STIL: Subtransverse process intraligamentary plane
CTF: Costotransverse foramen
MIC: Multiple injection costotransverse
ITP: Intertransverse process
